# Morphine Modulates Adult Neurogenesis and Contextual Memory by Impeding the Maturation of Neural Progenitors

**DOI:** 10.1371/journal.pone.0153628

**Published:** 2016-04-14

**Authors:** Yue Zhang, Chi Xu, Hui Zheng, Horace H. Loh, Ping-Yee Law

**Affiliations:** 1 Department of Pharmacology, University of Minnesota, 6–120 Jackson Hall, 321 Church St. S.E., Minneapolis, Minnesota 55455–0217, United States of America; 2 South China Institute for Stem Cell Biology and Regenerative Medicine, Guangzhou Institutes of Biomedicine and Health, Chinese Academic of Sciences, 190 Kaiyuan Ave, Guangzhou 510530, China; Xi'an Jiaotong University School of Medicine, CHINA

## Abstract

The regulation of adult neurogenesis by opiates has been implicated in modulating different addiction cycles. At which neurogenesis stage opiates exert their action remains unresolved. We attempt to define the temporal window of morphine’s inhibition effect on adult neurogenesis by using the POMC-EGFP mouse model, in which newborn granular cells (GCs) can be visualized between days 3–28 post-mitotic. The POMC-EGFP mice were trained under the 3-chambers conditioned place preference (CPP) paradigm with either saline or morphine. We observed after 4 days of CPP training with saline, the number of EGFP-labeled newborn GCs in sub-granular zone (SGZ) hippocampus significantly increased compared to mice injected with saline in their homecage. CPP training with morphine significantly decreased the number of EGFP-labeled GCs, whereas no significant difference in the number of EGFP-labeled GCs was observed with the homecage mice injected with the same dose of morphine. Using cell-type selective markers, we observed that morphine reduced the number of late stage progenitors and immature neurons such as Doublecortin (DCX) and βIII Tubulin (TuJ1) positive cells in the SGZ but did not reduce the number of early progenitors such as Nestin, SOX2, or neurogenic differentiation-1 (NeuroD1) positive cells. Analysis of co-localization between different cell markers shows that morphine reduced the number of adult-born GCs by interfering with differentiation of early progenitors, but not by inducing apoptosis. In addition, when NeuroD1 was over-expressed in DG by stereotaxic injection of lentivirus, it rescued the loss of immature neurons and prolonged the extinction of morphine-trained CPP. These results suggest that under the condition of CPP training paradigm, morphine affects the transition of neural progenitor/stem cells to immature neurons via a mechanism involving NeuroD1.

## Introduction

Addictive drugs such as opiates cause long-lasting changes in the brain, which influences many different forms of neural plasticity [1,2]. Among the multiple forms of neural plasticity mechanisms that contribute to drug memory, adult neurogenesis in the sub-granular zone (SGZ) of the dentate gyrus (DG) in the hippocampus has been implicated in drug reward and relapse due to the substantial roles that adult neurogenesis has in hippocampus function during learning and memory [3,4]. Several addictive drugs have been shown to alter adult neurogenesis. The psychomotor stimulants methamphetamine and cocaine decreased proliferation or maturation of hippocampal neural stem cells [5], and withdrawal from cocaine normalizes deficits in the proliferation of adult-born granular cells (GCs) [6]. Chronic morphine, administered via subcutaneous pellet implantation, was shown to decrease the number of proliferating cells in the SGZ in rodents; a similar effect was also observed in rats after chronic self-administration of heroin [7], while following extinction from heroin-seeking behavior, the formation of immature neurons in the DG was increased [8]. Conversely, a knock-out of the mu-opioid receptor was shown to enhance adult-born hippocampal GCs’ survival [9]. There are also reports suggesting that chronic morphine influences the neurogenic microenvironment in the DG by regulating certain growth factors [10]. In cultured neural progenitor cells, morphine treatment was shown to alter neural proliferation and differentiation, and it was also shown to promote apoptosis [11]. A recent study in our lab showed in detail that morphine exposure affects neurogenesis by modulating the cell-lineage in cultured neural stem cells [12].

Recent studies suggested that adult neurogenesis in the DG also has substantial roles in the drug addiction cycle. For instance, suppression of adult neurogenesis significantly increased cocaine self-administration [13]. Stress, a well-known factor that decreases adult neurogenesis, had significant positive relationships with ratings of craving for cocaine, heroin, and tobacco [14]. Some positive regulators of hippocampal neurogenesis such as environment enrichment and voluntary exercise, on the contrary, reversed learned drug associations in an animal model of cocaine-induced CPP [15], and they protected against methamphetamine self-administration [16]. These results suggest a negative correlation between drug addiction and level of adult hippocampal neurogenesis.

Because neural stem/progenitor cells (NSPCs) in the adult hippocampus could be divided into several types according to their different developmental stages, it is important to determine the specific types and developmental stages of NSPCs on which addictive drugs such as morphine exert their actions to further speculate about the potential mechanisms underlying the drug’s regulation. However, morphine’s negative effect on adult neurogenesis is largely dependent on the manner in which the drug was administered. Implantation of morphine pellets resulted in a negative effect on adult neurogenesis [7], while intraperitoneal injections of morphine failed to show any significant influence [17]. It was demonstrated that chronic morphine affected proliferation of type-2b and type-3 NSPCs but not others [18]. This discrepancy in morphine’s negative effect remains in a CPP paradigm, in which morphine injection decreased the number of neural progenitors in the DG labeled by Doublecortin (DCX) and other neurogenesis markers [19]. Furthermore, a transcriptional factor, neurogenic differentiation 1 (NeuroD1), was shown to participate in morphine’s regulation of adult neurogenesis. NeuroD1 is one of the conserved transcription factors that are expressed in the same order during glutamatergic neurogenesis in the developing cerebellum and in the adult dentate gyrus [20]. It has been shown to be involved in the differentiation of the progenitor cells and migration of immature neurons [21] (Von Bohlen and Halbach 2007). Several in vivo studies support the fact that NeuroD1 has an important role in specifying the neuronal fate during hippocampal neurogenesis [22], and it is essential for the survival and maturation of adult-born neurons [23]. Thus, a probable mechanism for morphine’s inhibition on adult neurogenesis is the drug regulation of progenitor cells’ differentiation of immature neurons via its control of NeuroD1 activity. Our recent in vitro studies in isolated hippocampal progenitor cells suggest that by regulating Prox1/Notch1 activities via its control of the miR181a level, morphine alters the cell lineage of the progenitor cells during differentiation, favoring the differentiation into glia instead of neurons [24]. Whether the in vivo effects of morphine are similar to in vitro observations remains to be demonstrated.

To delineate the precise mechanism in which morphine modulates adult neurogenesis, we utilized a transgenic animal model in which a subpopulation of newborn granule neurons of the DG was labeled by EGFP under the control of a pro-opiomelanocortin (POMC) promoter. EGFP expression at the SGZ is transient, peaking at two weeks after the radial glial (neural progenitor) cells’ mitosis and turning off by one month [25]. An enhancement of adult neurogenesis was found in these mice represented by an increase in EGFP-labeled newborn GCs in the DG region of hippocampus during a CPP behavior test paired with saline, but a morphine training CPP significantly blocks this effect. By classifying neural progenitors in different developmental stages on the basis of the morphology of EGFP+ cells and co-expression with other immunohistochemical markers that reflect different stages of neurogenesis, we were able to demonstrate that morphine modulates adult neurogenesis by interfering with the transition of NeuroD1-expressing type-II and type-III neural precursors into DCX and Tuj-1 expressing immature neurons. Furthermore, a decrease in co-localization between BrdU+ and DCX+ cells and an increase in co-localization between BrdU+ and GFAP+ cells were observed. Overexpression of NeuroD rescued the negative regulation of morphine on adult neurogenesis and strengthened the animal’s memory of the drug experiences. Thus, our current in vivo studies support our in vitro reports that morphine’s negative effect on adult neurogenesis is mainly due to the drug’s action on the transition of progenitors into immature neurons. The over-expression of NeuroD1 facilitates the transition, negating morphine’s effect on adult neurogenesis and the subsequent retention of drug experience.

## Materials and Methods

### Animals

Eight-week-old C57BL/6J-Tg (POMC-EGFP) 1Low/J male mice were purchased from Jackson Laboratories (Bar Harbor, ME) (http://jaxmice.jax.org/strain/009593.html). The transgene from which the mouse line was generated was described by Overstreet et al. [25]. Mice were mated and all offspring were genotyped as described in a protocol from Jackson Laboratory Genotyping Database (Stock number: 009593). Three-month-old male transgenic carriers were used in the behavior study. Animal maintenance and procedures were conducted according to the Institutional Animal Care and Use Committee (IAUCUC) policies at the University of Minnesota.

### Condition Place Preference (CPP)

**Fig 1:**
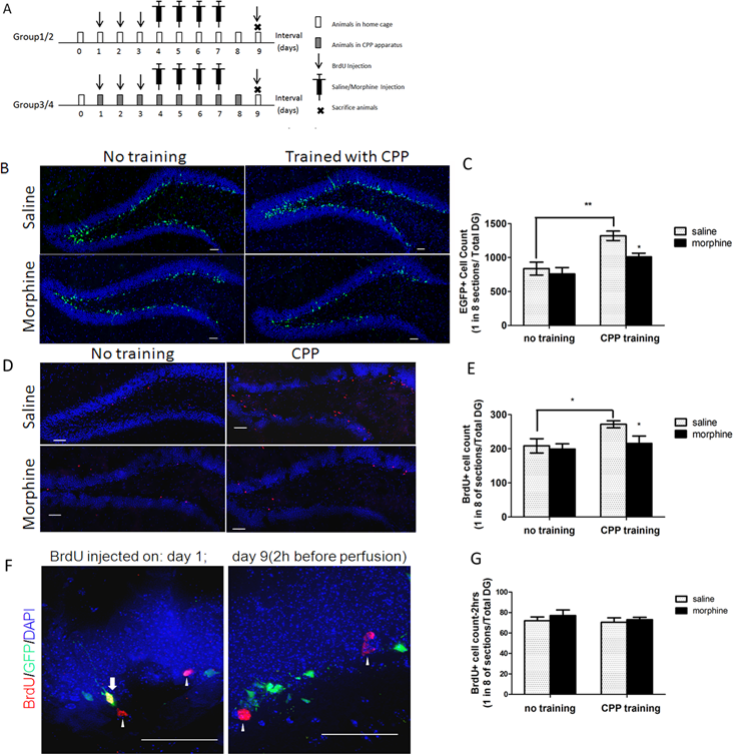
Morphine injection decreases adult-born DGCs labeled with EGFP only after going through a CPP paradigm. (A) Experiment design. On days 1–3, two groups of animals started habituating to the CPP apparatus and received 200 mg/kg BrdU i.p. injections once daily for three days, while the other two groups only received the BrdU injection. Pre-conditioning preference scores were recorded on day 3. From days 4 to 7, mice received saline injections in the morning and saline or morphine injections in the afternoon; after the injection, group 1/2 mice were put back in their homecage, while group 3/4 mice were trained in the CPP chamber for 30 min. After four days of training, group 3 and group 4 mice were tested for their post-conditioning preferences in a 15 min drug-free post-condition test (S1 Fig). On day 9, a subset of mice that did not receive the BrdU injections before were injected with BrdU one time 2 h prior to perfusion. All animals were sacrificed on day 9. (B) Comparison of the images of EGFP fluorescent from four groups of mice. (C) Fluorescent images of BrdU labeled newborn cells (Red) from four groups (Antigen-retrieval with acid often disrupted morphology of the granular layer). (D) Neural proliferation measured by average number of EGFP labeled newborn granular cells (count every one in eight brain sections, add together) in total dentate gyrus (N = 10/per group, *p<0.05, **p<0.01). (E) Neural progenitors’ survival was measured by the average number of BrdU (injected three days prior to CPP) labeled granular cells in the mouse dentate gyrus (N = 6/per group, *p<0.05). Data represent mean ± SEM of six to 10 animals in separate experiments. (F) Comparison of the fluorescent images of BrdU-labeled cells when injected on day 1–3 or day 9. (White arrow: BrdU-labeled cells colocalized with EGFP fluorescent; White arrowhead: BrdU-labeled cells did not colocalize with EGFP positive cells). (G) Neural proliferation measured by average number of BrdU (injected 2 h before perfusion) labeled granular cells in the mouse dentate gyrus (N = 4/per group, p>0.05). Statistical significance was determined by two-way ANOVA with a Bonferroni test for post hoc comparisons.

Mice stereotaxically injected with lentivirus were allowed to habituate for at least one week in a 12/12 h reverse-light-cycle room prior to the CPP paradigm. The experiment was carried out in a three chamber apparatus supplied by Panlab (Harvard Apparatus, Holliston, MA) according to the protocol [26] with a slight modification, as described previously [19]. Briefly, in the habituation stage (day 1–2), mice were allowed free access to all three chambers for 15 min per day. On day 3, mice’s place preference between two chambers (black and white) was tested and recorded as a pre-conditioning preference. Mice with strong bias to one of the chambers were rejected from the subsequent study. The rest of the mice were trained with saline or morphine for four days (Fig 1A), received a saline injection subcutaneously in the morning, spent 30 min in one chamber (black or white, randomly chosen), received either 5 mg/kg morphine or an equal volume of saline injection (s.c.) in the afternoon, and then spent another 30 min in the complementary chamber. On day 8, mice were tested for post-conditioning preference. During the extinction stage, the place preference of each mouse was tested once a week until no significant preference was detected in two continuous tests (S1 Fig). AnyMaze software (Stoelting, Wood Dale, IL) was used to track and record the position of mice in the apparatus during the testing period. The place preference of each individual mouse was calculated as follows:

CPP score=Time in drug-paired chamber(after training- before training)(second)

### Lentivirus construction

A Lentivirus V5-DEST construct expressing NeuroD1-cDNA was prepared as described previously [27]. The TRC Lentiviral shRNA of NeuroD (5’-CCGGGCTCAGCATCAATGGCAACTTCTCGAGAAGTTGCCATTGATGCTGAGCTTTTTG-3’) and a corresponding negative control construct were obtained from University of Minnesota Genomics Center. Virus particles were assembled in 293FT cells by transfecting V5-DEST constructs or shRNA constructs together with pLP1, pLP2 and pLP-VSVG constructs from the BLOCK-iT™ Lentiviral RNAi Expression System (Invitrogen, Grand Island, NY) following the manufacturer's instructions. Viral titers (~1.0 × 10^8^ TU/mL) were determined by assessment of DNA sequences in transduced 293FT cells [28]. NeuroD1 gene expression and knock down were tested by western blot analysis.

### Stereotaxic Injection

Stereotaxic injection of lentivirus was carried out as described [19]. Mice were anesthetized with isoflurane inhalation and a hole of 0.5 mm was drilled at 1.9–2.0 mm posterior to the bregma and ±1.0 mm lateral to the midline of each side. A needle of 28 gauges was connected with a flexible tube filled with virus and lowered 2.1 mm below the meniscus. Using a micro-syringe pump controller, 2 μl of virus (1x10^8^ TU/ml) was injected at a constant rate over a 5 min period. The virus was allowed to diffuse for 5 min and the needle was then raised slowly at a constant rate over a 2-min period. The holes were closed with bone wax and the wound on scull was sealed with Vetbond™ Tissue Adhesive (3M™, St. Paul, MN). Mice were allowed to recover for one week before any behavior study. As we have reported earlier, expression of NeuroD1 was observed in all cell types within the DG immediately after virus injection, but only distinct cells showed NeuroD1 expression 1 week after injection (Zheng et al, 2013). Such observation is in agreement with the literature report that only adult generated neural progenitors are target for lentivirus-mediated transgene delivery, 1 week after injection [29].

### Bromodeoxyuridine (BrdU) labeling and Immunohistochemistry

**Fig 2:**
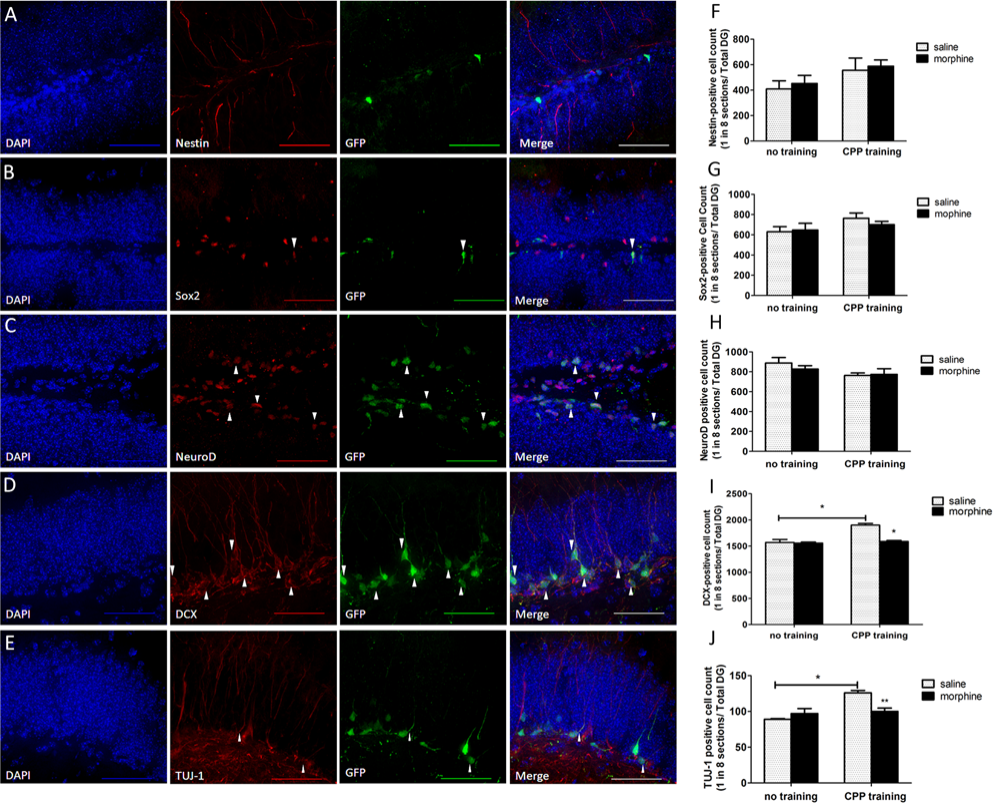
Morphine injections for four days decreased the number of later stage progenitors and immature neurons. (A-E) Fluorescent images of EGFP labeled cells and multiple neurogenesis markers: Nestin, Sox2, NeuroD, DCX, TUJ-1. There were no or very little Nestin or Sox2 positive neurons that were also EGFP positive (<5%), but there was a high percentage of NeuroD (20%-40%) or DCX (60%-80%) positive cells colocalized with EGFP neurons (colocalization marked by white arrow heads. Scale bar: 50 μm). (F-H) Measurement of the numbers of nestin, Sox 2 or NeuroD marked early neural precursors (N = 6/per group, no significant differences). (I-J) Measurement of the numbers of DCX or Tuj-1 labeled type III cells or immature neurons (N = 8/per group, *p<0.05). Statistical significance was determined by two-way ANOVA with a Bonferroni test for post hoc comparisons.

Mice were injected with BrdU (200 mg/kg i.p.) for three continuous days prior to CPP training (Fig 1A) or 2 h prior to perfusion. A subset of mice was sacrificed after CPP training, and the rest were maintained and tested until their place preference was totally extinct. Mice were deeply anesthetized with ketamine and xylazine and perfused with 0.9% saline followed by 4% paraformaldehyde. Brains were post-fixed with 4% paraformaldehyde, transferred into 10% sucrose and 30% sucrose sequentially for dehydration and then embedded in an O.C.T. compound. 30 μm coronal sections were cut through the dentate gyrus of the hippocampus (-0.82 to -3.70 mm post bregma) with a cryostat and every one in eight sections were mounted on the same Superfrost plus glass slides (Thermo Fisher Scientific, Waltham, MA) for future analysis. Thus, there were approximately 10 to 12 sections on the same slide, and any two of them were 0.24 mm apart in the stereotaxic Y-axis of hippocampus (S4 Fig).

For immunohistochemistry, mouse coronal sections were rinsed with TBS three times, blocked with 5% goat serum or donkey serum (with 1.5% BSA in TBS) at room temperature for 1 h and then incubated with the primary antibody. An extra antigen retrieval step was needed for BrdU and NeuroD1 staining prior to serum blocking: sections were treated with 1 M HCl at 40 ˚C for 30 min, and then they were incubated with 10 mM of Boric Acid for 10 min and washed with TBS for 5 min. The following primary antibodies were used in our studies:

Mouse monoclonal anti-GFP (1:2000; Life technologies, Grand Island, NY), Rat anti-BrdU (1:800; Abcam, Cambridge, MA), Mouse monoclonal anti-nestin (1:500; Abcam), Rabbit anti-Sox2 (1:500; Abcam), Rabbit DCX antibodies (1:1000; Abcam), Goat NeuroD antibodies (1:400; Santa Cruz Biotechnology, Santa Cruz, CA), Mouse monoclonal anti-Tuj1 IgG (1:1000; Covance, Princeton, NJ), Rabbit anti-GFAP (1:1000, Dako, Carpinteria, CA), Rabbit Caspase-3 antibodies (1:1000, Cell Signaling, Danvers, MA); Rabbit MOR antibodies (1:1000; Genentech, Customer ordered; San Francisco, CA)

For the TUNEL Assay, we use Click-iT^®^ Plus TUNEL Assay for *in situ* apoptosis detection with the Alexa Fluor^®^ dyes kit (Life technologies, Grand Island, NY; Catalog number: C10618) following the manufacturer’s instructions.

Secondary antibodies were used as follows:

Alexa Fluor 488 goat anti-mouse IgG, Alexa Fluor 594 goat anti-rabbit IgG, Alexa Fluor 594 goat anti-rat IgG, Alexa Fluor 647 goat anti-rabbit IgM, Alexa Fluor 594 donkey anti-goat IgG, Alexa Fluor 647 donkey anti-goat IgG (Life technologies). The dilution of all secondary antibodies is 1:2000.

### Quantification and Statistic Analysis

Coronal sections were mounted with DAPI Fluormount G (Sigma-Aldrich, St. Louis, MO) and visualized using a Leica DFC365 FX 1.4 mp monochrome digital camera connected to a Leica DM5500 B upright microscope (Leica, Germany). Leica Application Suite (Leica, Germany), Metamorph (Sunnyvale, CA) and ImageJ (NIH, MA) software were used to perform cell counting, fluorescent intensity, and co-localization analysis of images. To quantify the total number of fluorescent-positive cells in the SGZ, every 8th sections was mounted on the same slide, and the images of every section on the same slide throughout the dentate were captured (10–12 sections in total). The number of marker positive cells from each section were counted by the cell-counting program ImageJ and added together to represent the number of marker positive cells of one animal, and then the average summation of every animal in one group was used to represent the expression level of one neurogenesis marker. For the colocalization analysis, the images of every 8^th^ sections was captured, and all the cells with double positive fluorescent (e.g., BrdU+ in red and GFP+ in green, merged in yellow) in the dentate gyrus (DG) area were counted, divided by the total number of BrdU positive cells. The stereotaxic quantification from the front to the back through the hippocampus (bregma -0.82mm to -3.46mm) was also provided to represent the average distribution of these marker positive cells (6–8 animals), Every coronal section is 0.03mm in thickness, so the intervention between two adjacent samples is 0.24mm, as shown in S4 Fig.

Statistical analysis was performed with the GraphPad Prism 5.0 software. Statistical significance was determined by a student t-test, one-way or two-way ANOVA with a Bonferroni test for post hoc comparisons.

## Results

### CPP paradigm is essential for a morphine-induced decrease in POMC-EGFP-expressing neurons

In transgenic mice, the EGFP-expressing cells in the sub-granular zone (SGZ) of the dentate gyrus represent adult-born neurons, which are 3–28 days post-mitotic [25]. Four groups of male POMC-EGFP transgenic mice received saline (0.9% NaCl) or morphine (5 mg/kg) subcutaneous injections, a.m. and p.m., respectively. Two groups of mice only received injections in their home cages (Group 1/2), while the other two groups went through a CPP training paradigm (Group 3/4) (Fig 1A). On day 8, these two groups of mice were tested for CPP preferences (S1 Fig). Immunohistochemistry analysis showed that there was a significant difference in the number of green fluorescent cells in the dentate gyrus between the saline group (1319±69.1; N = 10) and the morphine group (1010±54.0; N = 10) after CPP training, but there was not a significant difference in the two groups without training (saline: 930±18.8; morphine: 758±94.2; N = 10) (Fig 1D; two-way ANOVA, effect of training: *F*_*1*,*36*_ *= 21*.*16*,*p<0*.*01*; effect of drug: *F*_*1*,*36*_ *= 5*.*91*, *p<0*.*05*) (Fig 1B).

When the S-phase marker BrdU was used to label the proliferating cells right before mice were exposed to morphine or saline, similar results were obtained. A subset of animals from the four groups received three injections of BrdU (200mg /kg, i.p.) three days prior to the CPP training (day 1-day 3), while the rest of the animals received only one injection 2 h prior to perfusion (day 9) (Fig 1A). For mice injected with BrdU during day 1 to 3, the groups trained with morphine show a significant decrease in the number of BrdU labeled cells in their SGZ (saline: 272±10.3; morphine: 215±21.6; n = 6), but no significant difference was found in groups without training (saline: 209±20.8; morphine: 199±15.3; n = 6) (Fig 1E; two-way ANOVA, effect of training: *F*_*1*, *20*_ *= 5*.*102*, *p<0*.*05*; effect of drug: *F*_*1*, *20*_ *= 3*.*463*, *p<0*.*05*) (Fig 1C). However, for mice injected with BrdU on day 9, 2 h prior to perfusion, no significant differences in the number of BrdU-labeled cells were detected between the four groups (Fig 1G). Comparing the fluorescent images of BrdU labeled cells under the microscope, we found that 50%-60% of the BrdU-labeled cells were also EGFP positive (Fig 1F, left panel) from mice injected with BrdU on days 1–3, while there was no colocalization between BrdU and EGFP positive cells (Fig 1F, right panel) from mice injected with BrdU on day 9. Our observation supported the former report that in this animal model, EGFP labeled adult-born neurons were 3–28 days post-mitotic [25], but they were not early proliferating neural stem cells.

These results indicated that CPP training increases the number of neural progenitors labeled by EGFP and enhanced the survival of adult-born neurons, but this effect was blocked by morphine treatment.

### Morphine decreased the number of late stage progenitors and immature neurons after CPP training, but not early type-I, type-II progenitors in SGZ

Further immunohistochemistry analysis showed that neither morphine injection in the home cage nor that during CPP training changed the numbers of early stage of NSPCs. Mice from different groups have no significant differences in the number of proliferating cells, as shown by the S-phase marker BrdU (Fig 1G) or cellular proliferating marker Ki-67 (S2 Fig). Additionally, the four groups of transgenic mice showed similar levels of early neurogenesis marker expression, including nestin (Fig 2F), Sox2 (Fig 2G) or NeuroD1 (Fig 2H) labeled type I and type II cells (Fig 2A–2C).

On the other hand, mice trained with morphine in CPP showed a decreased number of both DCX (Fig 2I; two-way ANOVA, effect of training: *F*_*1*,*20*_ *= 28*.*15*, *p<0*.*05*; effect of drug: *F*_*1*,*20*_ *= 22*.*69*, *p<0*.*05*) (Fig 2D) and Tuj-1 (Fig 2J; two-way ANOVA, effect of training: *F*_*1*,*20*_ *= 19*.*05*, *p<0*.*01*; effect of drug: *F*_*1*,*20*_ *= 3*.*857*, *p<0*.*05*) (Fig 2E) labeled type III cells or immature neurons. No change in the DCX or Tuj-1 labeled cells was found in groups without CPP training (DCX: saline-no training: 1604±54.8; morphine-no training: 1588±24.2; saline-CPP: 1836±38.5; morphine-CPP: 1596±18.2; N = 6) (Tuj-1: saline-no training: 79±4.1; morphine-no training: 98±11.2; saline-CPP: 126±6.3; morphine-CPP: 97±8.5, N = 6). In a previous study, we reported that morphine decreased NeuroD1 activity but not the NeuroD1 protein level in rat hippocampal primary neuron cultures [27]. A similar morphine effect was now observed *in vivo*. In the hippocampus from the transgenic mouse trained with morphine in the CPP paradigm, we failed to detect a significant difference in the numbers of NeuroD1-labeled neural progenitors (Fig 2H); instead we observed a decrease in the numbers of DCX-labeled neural progenitors in the group trained with morphine compared to saline (Fig 2I). Since DCX is one of the transcriptional targets of NeuroD1, such results indicate NeuroD1 activity was affected. These data imply that, compared to its direct influence on early proliferation, morphine is more likely to interfere with certain late stages of development of adult-born neural progenitors.

Our conclusion is further supported by morphology analysis because morphine training increases the ratio of NSPCs in an oval shape and without noticeable neurite growth, while in the meantime, it decreases the ratio of NSPCs with a long or branching dendrite protruding inner molecular layer (S3 Fig). To confirm the change in cell numbers with a quantification throughout the entire hippocampus, we added the stereotaxic quantification for each neurogenesis marker mentioned in result 3.1 and 3.2 (S4 Fig).

### Morphine changed the lineage of neural progenitors’ differentiation

**Fig 3:**
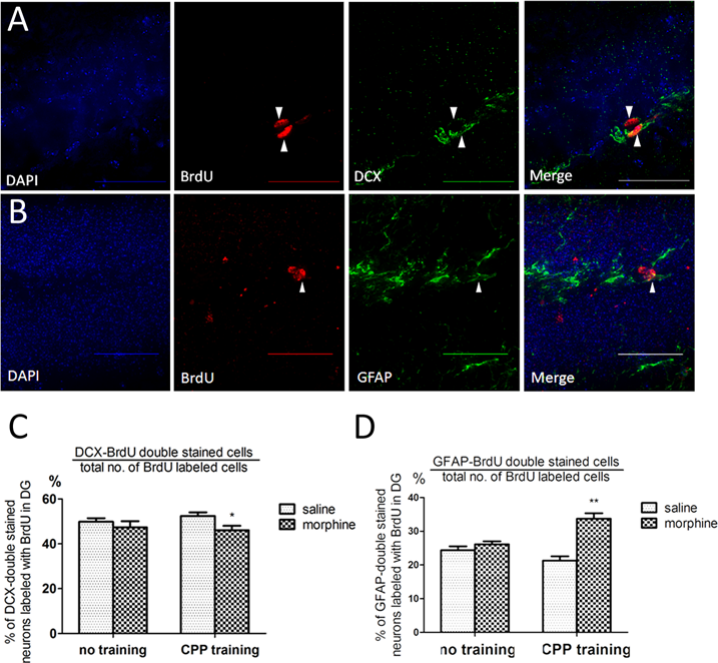
Morphine decrease co-localization between BrdU-DCX labeled immature neurons, while increase co-localization between BrdU-GFAP labeled glia. (A-B) Fluorescent images of BrdU labeled cells colocalized with immature neuron marker DCX and glia marker GFAP (colocalization marked by white arrow heads. Scale bar: 50 μm). (C) Measurement of the colocalization between DCX and BrdU between groups (N = 6/per group, *p<0.05). (D) Measurement of the colocalization between GFAP and BrdU between groups (N = 6/per group, **p<0.01). Data represent mean ± SEM of six to eight animals in separate experiments. Statistical significance was determined by two-way ANOVA with a Bonferroni test for post hoc comparisons.

After analyzing the lineage of surviving progenitors labeled by BrdU after injection from day 1 to day 3 (Fig 1A), we observed a decrease in the co-localization between DCX and BrdU-labeled neurons 9–12 days postmitotic (Fig 3C; two-way ANOVA, effect of training: *F*_*1*, *20*_ *= 0*.*11*, *p = 0*.*75*; effect of drug: *F*_*1*, *20*_ *= 4*.*74*, *p<0*.*05;* N = 6) (Fig 3A). In the meantime, there was an increase in the co-localization between GFAP and BrdU-labeled newborn glia (Fig 3D; two-way ANOVA, effect of training: *F*_*1*, *20*_ *= 2*.*95*, *p = 0*.*10*; effect of drug: *F*_*1*, *20*_ *= 29*.*10*, *p<0*.*01;* N = 6) (Fig 3B). These observations are consistent with our previous report showing that in cultured neuron stem cells, morphine decreases the percentage of immature neurons such as beta-tubulin 3(TUJ-1) positive cells in neural stem cell cultures, but it increases the percentage of GFAP-expressing glia [24].

**Fig 4:**
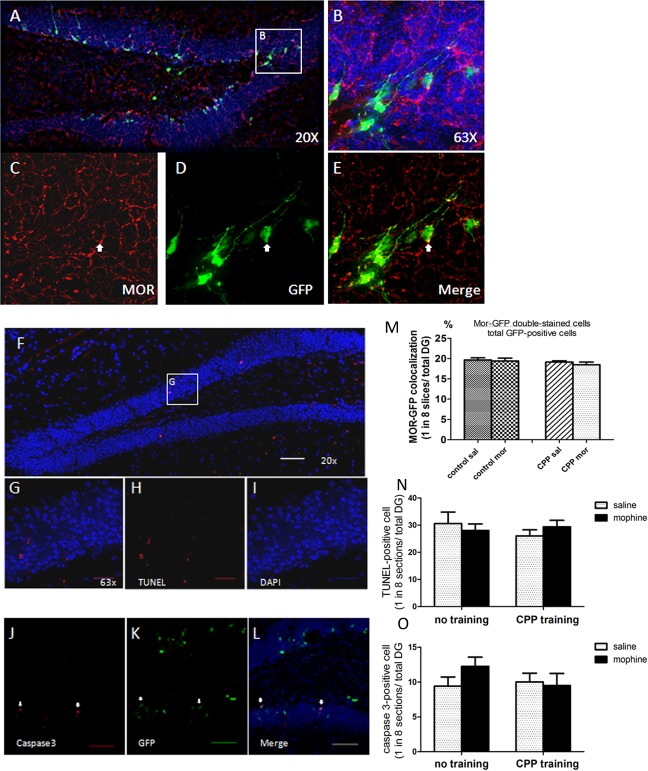
Expression of the Mμ-opioid-receptor protein (MOR) and apoptosis in the dentate gyrus of the hippocampus. (A-B) Photo image of MOR staining in the dentate gyrus of the hippocampus (Red: MOR; Green: GFP; Blue: DAPI; A. 20x objective lens, 200x magnification. scale bar: 50 μm; B. 630x magnification, oil-lens). (C-E) Fluorescent images of DG cells stained for GFP (Green) and MOR (Red). White arrow indicates GFP labeled cells colocalized with MOR. (F-I) Fluorescent images of DG cells stained for apoptosis (Red: TUNEL–positive, Blue: DAPI; F. 20x objective lens, 200x magnification. scale bar: 50 μm; G-I. 63x oil-lens, scale bar: 20 μm). (J-L) Fluorescent images of DG cells stained for apoptosis (Red: caspase 3, Green: GFP, Blue: DAPI; 20x objective lens, 200x magnification. scale bar: 50 μm). (M) Analysis of the colocalization between EGFP and MOR double-staining between groups. Control groups received no training, only injections; CPP groups were trained as in Fig 1A (N = 6, no significant differences). (N-O) Measurement of the numbers of TUNEL+ or Caspase3+ apoptosis cells (N = 6, no significant differences).

How did morphine decrease the number of immature neurons? Since a subset of the neural progenitors in the SGZ started to express the μ-opioid-receptor (MOR) a few days after birth (Fig 4A–4E), one probable mechanism could be that the activation of MOR in these progenitors resulted in apoptosis. If this hypothesis is correct, there should be less EGFP+ cells that are also MOR+ in the morphine-trained group than the saline-trained group. However, we observed that morphine did not alter the percentage of EGFP+ cells colocalized with MOR in the total EGFP-labeled neurons (Fig 4M), which suggested that the activation of MOR on the surface of the immature neurons by morphine did not lead to the reduction of EGFP+ cells. This conclusion is confirmed by TUNEL-assay (Fig 4F–4I) and Caspase-3 staining (Fig 4J–4L) of SGZ, in which a significant difference in the number of apoptosis cells between the saline and morphine groups was not detected (Fig 4N and 4O). These data indicated that morphine exerts its effect on hippocampal neural progenitors by increasing the immature NeuroD1-expressing neuron level and immature glia level, meanwhile decreasing the more mature DCX-expressing neuron level. Such effects of morphine on cell lineage did not involve a mechanism of inducing apoptosis.

### NeuroD1 over-expression in the hippocampus by lenti-viral injection restored the population of immature neurons and prolonged the extinction phase of morphine-induced CPP

**Fig 5:**
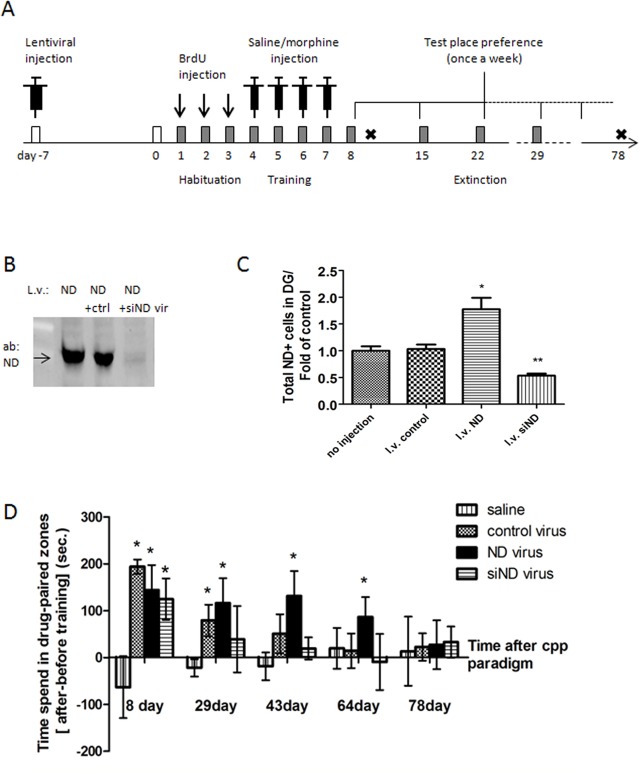
Overexpression or knock down of NeuroD protein in the system. (A) Experiment design: Lenti-virus of control oligonucleotide (control), NeuroD cDNA (ND) or NeuroD siRNA (siND) was injected one week prior to the CPP study. A subset of mice from the control and ND groups was sacrificed on day 9 for the following staining analysis. (B) Western blot test of the NeuroD virus *in vitro*. (C) Immunoactivity test of the NeuroD and NeuroD siRNA virus *in vivo*. The NeuroD cDNA virus increases the total number of NeuroD expressing cells in the DG by 1.8-fold compared to the no-injection control, while NeuroD siRNA decreases the number of NeuroD expressing cells in the DG to 0.53-fold of the control (N = 4,*p<0.05, **p<0.01). (D) The CPP extinction test measured by time spent in the drug-paired chamber in different groups (N = 6–8, *p<0.05). On day 8, all groups injected with the virus developed CPP to the same extent. However, the group injected with siRNA of NeuroD lost their drug-chamber preference on day 29, while the group injected with the control virus and NeuroD virus lost their preference on day 43 and 78, respectively.

Our previous studies have indicated that NeuroD1 is one of the essential targets during morphine’s regulation of adult neurogenesis. To examine whether NeuroD1 activity is the basis for morphine’s negative effect on the EGFP+ cells, we established lentiviral constructs that expressed cDNA of NeuroD1, a small-interfere RNA specifically targeting NeuroD1, and an empty viral vector. Lentivirus was stereotaxically injected into the transgenic mouse hippocampus before the CPP paradigm (Fig 5A). We tested lentiviral gene expression by transducing three groups of viruses in the HEK293T cell line, which has no endogenous NeuroD1 expression (Fig 5B). We also tested NeuroD1 expression *in vivo* by delivering lentivirus in the mouse hippocampus (Fig 5C, One way ANOVA, *F = 19*.*40*, *p<0*.*05;* N = 4). In the subsequent CPP study, we found that mice injected with NeuroD1 siRNA showed the shortest period of extinction phase, and their place preferences related to drug memory were gone (comparing pre- and post-conditioning preference) within 29 days after acquisition (Fig 5D). The place preference of drug lasted for 43 days for mice that received the control virus and approximately 78 days for mice that received the NeuroD1 virus (Fig 5D; student t-test comparison, p<0.05; N = 6–8). The magnitude of place preference and the length of extinction days after training were almost identical between groups of mice that received the control virus injection and mice that did not have a stereotaxic injection (S1 Fig), indicating that the surgery did not impair mice’s ability to form and retain drug-memory.

**Fig 6:**
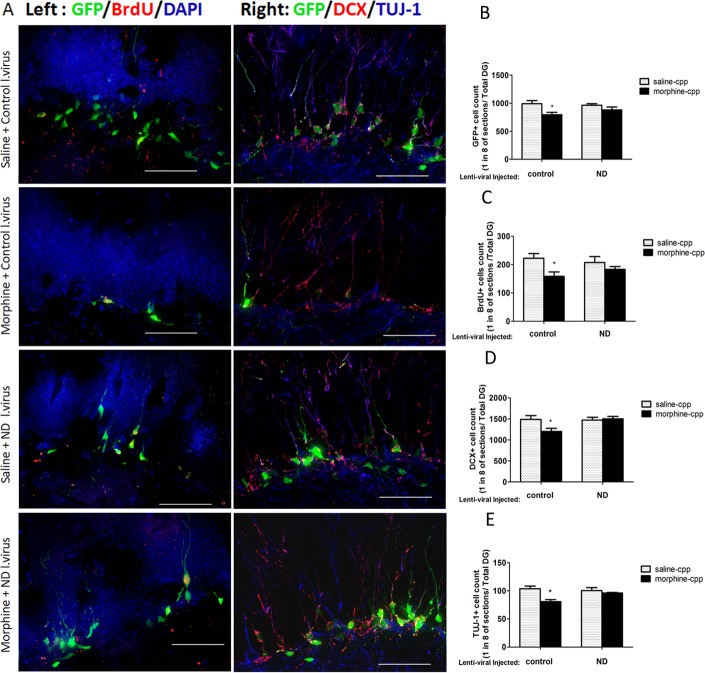
Overexpression of NeuroD by injection of lenti-virus in mouse hippocampus. (A) Fluorescent images of four groups of mice (Fig 5A, sacrificed on day 9) that received different lenti-virus injections (control/ND) and CPP training (saline/morphine injection). Left panel: GFP-BrdU double staining; Right panel: DCX-Tuj-1 double staining (scale bar: 50 μm). (B-C) Measurement of the numbers of EGFP and BrdU labeled neural precursors (N = 8, *p<0.05). (D-E) Measurement of the numbers of DCX and Tuj-1 labeled immature neurons (N = 6, *p<0.05). Data represent mean ± SEM of 6–8 animals in separate experiments. Statistical significance was determined by two-way ANOVA with a Bonferroni test for post hoc comparisons.

When we examined the brain sections of mice that were injected with the control or NeuroD1 virus and subjected to the CPP paradigm, we confirmed that morphine’s negative effect on adult neurogenesis could be restored by the overexpression of NeuroD1. The decrease in numbers of EGFP-labeled adult-born granular cells was detected in the control-virus group (saline-CPP: 991±56.1; morphine-CPP: 795±42.4; N = 8, p<0.05) but not in NeuroD1-virus group (saline-CPP: 963±29.5; morphine-CPP: 879±53.8; N = 8, p>0.05) (Fig 6B; two-way ANOVA, effect of virus: *F*_*1*, *28*_ *= 0*.*36*, *p = 0*.*55*; effect of drug: *F*_*1*, *28*_ *= 8*.*972*, *p<0*.*05*). Similarly, the difference in the survival of BrdU-labeled neural progenitors was observed in the control-virus group (saline-CPP: 223±18.2; morphine-CPP: 158±17.1; N = 8, p<0.05), but not in the NeuroD1-virus group (saline-CPP: 207±21.1; morphine-CPP: 183±10.3; N = 6, p>0.05) (Fig 6C; two-way ANOVA, effect of virus: *F*_*1*, *24*_ *= 0*.*083*, *p = 0*.*78*; effect of drug: *F*_*1*, *24*_ *= 7*.*057*, *p<0*.*05*) (Fig 6A). Furthermore, we also found that the number of DCX (Fig 6D; two-way ANOVA, effect of virus: *F*_*1*, *24*_ *= 5*.*658*, *p<0*.*05*; effect of drug: *F*_*1*, *24*_ *= 4*.*613*, *p<0*.*05*) and Tuj-1 (Fig 6E; two-way ANOVA, effect of virus: *F*_*1*, *24*_ *= 2*.*164*, *p = 0*.*16*; effect of drug: *F*_*1*, *24*_ *= 11*.*67*, *p<0*.*05*) labeled immature neurons decreased in the control-virus group (DCX: saline: 1493±78.5; morphine: 1275±64.2; Tuj-1: saline: 106±6.8; morphine: 82±5.2; N = 8, p<0.05) the same as in naive mice without the lentivirus injection (Fig 2), and the decrease was compensated in the NeuroD1-virus group (DCX: saline: 1487±54.9; morphine: 1517±49.1; Tuj-1: saline: 98±7.1; morphine: 94±2.6; N = 6, p>0.05) (Fig 6A).

These results indicated that morphine’s regulation on adult neurogenesis was dependent on NeuroD1 activity, and the overexpression of NeuroD1 could compensate for morphine’s negative effect on neurogenesis, but it could not enhance neurogenesis when compared to the control-virus group. In conclusion, morphine indirectly deactivated the transcriptional factor NeuroD1, which controlled a key step of neural progenitor differentiation in hippocampal neurogenesis, resulting in a reduction of the early progenitors differentiating into immature neurons.

## Discussion

### Morphine exerts actions on adult neural stem/progenitor cells (NSPCs) in the pre-differentiation stage

Adult neurogenesis is a multi-step dynamic process that is manipulated by many intrinsic and extrinsic factors. The neuron stem cells are defined as primary multifunctional progenitor cells that resemble radial glia and are characterized by the expression of GFAP and nestin, which are known as Type-I or B-type cells [30]. Type-I cells are thought to divide to produce intermediate stage progenitors (Type-II, Type-III or D-type cells), which then undergo further rounds of cell division to generate post-mitotic immature granule neurons [31]. Previously, we reported that opioid agonists such as morphine and fentanyl differentially regulated adult neurogenesis by altering NeuroD1 activity differently [19]. Although both fentanyl and morphine produced similar CPP responses after 4 days of training, only morphine treatment resulted in a decrease in adult neurogenesis[19,27]. So the effect on neural progenitors is morphine related, but not just related to learning a context-association.

NeuroD1 is one of the classic B basic Helix-Loop-Helix transcriptional factors that promotes premature cell cycle exit and differentiation in neural precursor cells [32]. NeuroD1 has been reported to be essential for late-stage progenitors to differentiate into hippocampal granule neurons [23], and it exhibits a pronounced neuron-inductive effect that leads to a granule neuron commitment [22]. Hence, by regulating NeuroD1 activities, opiate agonists such as morphine can modulate the newborn granular neurons’ differentiation into mature neurons.

The POMC-EGFP transgenic model allows the visualization of adult-generated granular cells in a restricted time window. Thus far, our study has demonstrated that the analgesic dose (5 mg/kg) of morphine injection during a contextual memory behavior task can regulate adult neurogenesis, and morphine probably interfered with the late phase of development of neural precursors rather than affecting the early proliferation stage. Our current study shows that CPP training alone is sufficient to enhance adult hippocampal neurogenesis, as shown by increased EGFP and BrdU labeled cells (Fig 1). This observation is supported by many behavior studies demonstrating that both physical exercise and environmental enrichment can increase adult neurogenesis [33–35]. Morphine treatment, on the other hand, blocks such enhancement by impeding the differentiation of early neural progenitors (type-I and type-II cells) into more mature stages of development (type-III and immature neurons), which are stages that are known to have NeuroD1 expressed [20, 36]. However, this process did not lead to an accumulation of NeuroD1 expressing progenitors because a large portion of these progenitors die within a few days following their birth [37]. It is reasonable to assume that the neural precursors that fail to differentiate into functional immature neurons would go through apoptosis. The massive cell death of adult-born granular neurons may serve as a natural selective mechanism since it has been demonstrated that cell survival and death are both important during learning and memory [38]. However, our TUNEL and Caspase-3 staining did not show morphine accelerating apoptosis in the dentate gyrus area (Fig 3).

An alternative explanation for the negative effect on adult neurogenesis is that morphine alters the fate of early progenitors indirectly, inducing more neural stem cell differentiation into glia rather than neurons. Our recent *in vitro* study with the isolated progenitor cells from the mouse hippocampus indicates that morphine alters the lineage of the progenitors during differentiation by regulating the Prox1/Notch1 pathway via the drug’s control of miR-181a [24]. Similar results were observed with our current *in vivo* study. Among all BrdU-labeled cells, morphine increased BrdU/GFAP co-localization and decreased BrdU/DCX co-localization (Fig 2). Whether morphine regulation of the Prox1/Notch1 pathway *in vivo* also contributes to the eventual decrease in the newborn progenitors differentiated into immature neurons during the CPP paradigm remains to be determined.

Our conclusion is further supported by the observation that the ratio of EGFP+/MOR+ cells was not altered by morphine (Fig 3) and that the over-expression of NeuroD1 could rescue the decrease in EGFP+ cells in the DG (Fig 6). If the morphine action were via the inhibition of early progenitor’s proliferation, then one would expect a decrease in the presence of MOR in the EGFP+ cells that survived. This was not the case. Because NeuroD1 activities are normally observed in the intermediate stages of progenitors, type II and type III cells, an increase in NeuroD1 expression should not rescue the morphine inhibitory effect if the drug’s action was on the regulation of early progenitors’ proliferation. Our previous studies indicated that morphine inhibited the CaMKIIα activity, thus attenuating the phosphorylation and activation of NeuroD1 [27]. The ability of NeuroD1 over-expression to block the morphine decrease in a number of EGFP+ cells in the SGZ suggested that one of morphine’s actions is via the regulation of NeuroD1-mediated maturation of the newborn granular neurons.

### Correlation between drug-experienced memory and the levels of hippocampal neurogenesis

Our study also indicates that morphine-induced contextual memory is related to the manipulation of adult hippocampal neurogenesis. When over-expressing NeuroD1 in the dentate gyrus to counteract morphine’s inhibition on adult neurogenesis, the animal exhibited much longer memory of their drug experience, which was represented by a prolongation of the CPP extinction time. On the other hand, when knocking down NeuroD1 with a RNA interference method, it has an opposite effect. These results suggest that the progenitors at a certain stage of development may be associated with contextual memory formation. This observation is concurrent with the reports that adult-born granule cells are more preferential in their incorporation into spatial memory networks in the dentate gyrus [39]. In addition, it is reported in the literature that reactive glial cells in the cortex of stab-injured or Alzheimer's disease (AD) model mice can be directly reprogrammed into functional neurons in vivo using retroviral expression of NeuroD1 [40]. Thus, it is also possible that NeuroD1 overexpression enhances adult neurogenesis and prolongs animal’s contextual memory by reprogramming of glial cells into functional neurons.

However, morphine’s negative effect on modulating the differentiation and maturation of neural progenitors may not be considered to be the only mechanism that affects an animal’s drug experienced memory. Our lab has also reported that morphine and fentanyl have different effects on dendritic spine stability through different modulations of NeuroD1 activity [27]. Such synaptic changes are considered essential for memory formation and retention [41]. A genome-wide prediction of NeruroD1 direct target genes includes multiple Eph receptors [42], which are reported as key players in synapse formation and plasticity [43]. Therefore, the fact that NeuroD1 overexpression prolonged morphine-induced CPP could be due to its effects in maintaining spine stability and synapse formation. How to distinguish the contribution of the synaptic effect of NeuroD1 on an animal’s drug memory formation from the contribution of its influence on progenitors’ development and fate choice in adult neurogenesis needs to be further established.

### Possible molecular mechanisms of morphine’s modulation and future prospects

In our previous study, we reported that the extinction of the fentanyl-trained CPP response could be shortened by the injection of the miR-190 lentivirus within the DG [19]. We surmised that such a miR-190 effect was due to its decrease in the NeuroD1 level. However, miR-190 has multiple targets, one of which is Pax6 that is associated primarily with the Type 1 progenitors and directly activates the *Neurogenin2 (Ngn2)* gene [44]. To confirm our initial observation that NeuroD1 activity is the key to the extinction of drug associated memory, we re-examined the extinction of morphine-associated memory by using siRNA to specifically decrease the NeuroD1 activity. Similar to previously reported observations, over-expressing NeuroD1 in the DG counteracted morphine’s inhibition on adult neurogenesis and extended the drug-associated memory (Fig 5D). In contrast, the expression of the NeuroD1 siRNA clearly shortened the extinction of the morphine-associated memory and decreased the number of ND+ cells in the SGZ (Fig 5C). These results clearly indicated the central role NeuroD1 exhibits in adult neurogenesis and subsequent drug-associated memory. Some studies indicated that NeuroD1 could be the upstream activator of the Pax6 gene and a downstream target of Ngn1 and Ngn2 [45]. These transcriptional factors are able to define a cascade with two other factors, T-box brain genes 1 and 2 (Tbr1 and Tbr2), in a sequence of Pax6-Ngn2-Tbr2-NeuroD-Tbr1, which plays a crucial role in regulating the adult SGZ NSPCs [46,47]. Furthermore, one of NeuroD1’s targets, Notch1, is known to up-regulate Hes and Herp [48], which are basic helix-loop-helix (bHLH) transcription factors that antagonize proneural genes such as Mash1 and Ngn [49]. Cells with higher Notch1 levels will continue to divide, while those with low levels differentiate [50]. Hence, whether the effects of morphine on adult neurogenesis are due to its control of this closed, positive-feedback loop of gene regulation (i.e., Pax6→Ngn2 →NeuroD1→Pax6) at the NeuroD1 level only or at multiple points of this gene circuit needs to be investigated.

However, it is clear that the morphine control of adult neurogenesis and subsequent contextual memory can trace back to the agonist regulation of various miRNAs. Activation of MOR by morphine resulted in an elevation of the miR-181a level [24], while the miR-190 level was not altered (Zheng, 2010; Zheng, 2013). Such differential regulation of the miRNA resulted in changes in the activities of the transcription factors that are associated with neurogenesis such as Prox1, Notch1 and NeuroD1. If the miRNA(s) central to the regulation of these transcription factors’ activities can be identified, one would expect that adult neurogenesis’ contribution to drug-associated memory could be manipulated by altering the miRNA(s) level(s) and subsequently the drug relapse. Whether such a scenario can be achieved remains to be determined.

## Supporting Information

S1 Fig (A) Prelimenary CPP test. WT mouse trained by morphine for 3 days started to show place preference. When trained for 4 days, the amplitude of CPP reach maximum. (B) On day 8 (Fig 1A), group 4 mice injected with 5mg/kg morphine acquired strong place preference to drug-paired chamber (N = 6-8/per group). (C) The WT mouse, POMC-EGFP transgenic mouse and mouse injected with control virus did not show any significant differences in CPP training and extinction (N = 6-8/per group). (D) Measurement of CPP extinction for 3 groups of mouse injected with different lentivirus.
(TIF)

S2 Fig (A) Fluorescent images of EGFP labeled cells and cell proliferation marker ki-67 (colocalization marked by white arrows. Scale bar: 50μm). (B) Measurement of the numbers of ki67 positive neural progenitors in SGZ (N = 4-6/per group, no significant differences).
(TIF)

S3 Fig (A) Example of EGFP-labeled granular cells with different morphology in dentate gyrus: ① progenitors without noticeable neurite growth; ② progenitors with short dendrite (single dendrite did not reach molecular layer) ③ progenitors with long dendrite (dendrite reached inner molecular layer (IML) or with branching) ④ progenitors migrate into granular cell layer (GCL). (B) EGFP-labeled cell morphology analysis; measured by percentage of each defined group of progenitors in total number of EGFP+ cells (N = 6/per group, *p<0.05). Mice trained with morphine showed more percentage of cells without noticeable neurite while less percentage of cells with long or branching dendrite. This data support our conclusion that morphine decelerate the maturation process of newborn granular neurons. Data represent mean ± SEM of 6 to 10 animals in separate experiments. Statistical significance was determined by two-way ANOVA with Bonferroni test as post hoc comparisons.
(TIF)

S4 Fig **(A-I)** Stereotaxic quantification for each neurogenesis marker mentioned in Figs 1 and 2.
(TIF)
