# Supplementary figures and images for: Morphine Modulates Adult Neurogenesis and Contextual Memory by Impeding the Maturation of Neural Progenitors
Source: PLoS One. 2016 Apr 14;11(4):e0153628. doi: 10.1371/journal.pone.0153628 (PMC4831694; doi:10.1371/journal.pone.0153628)

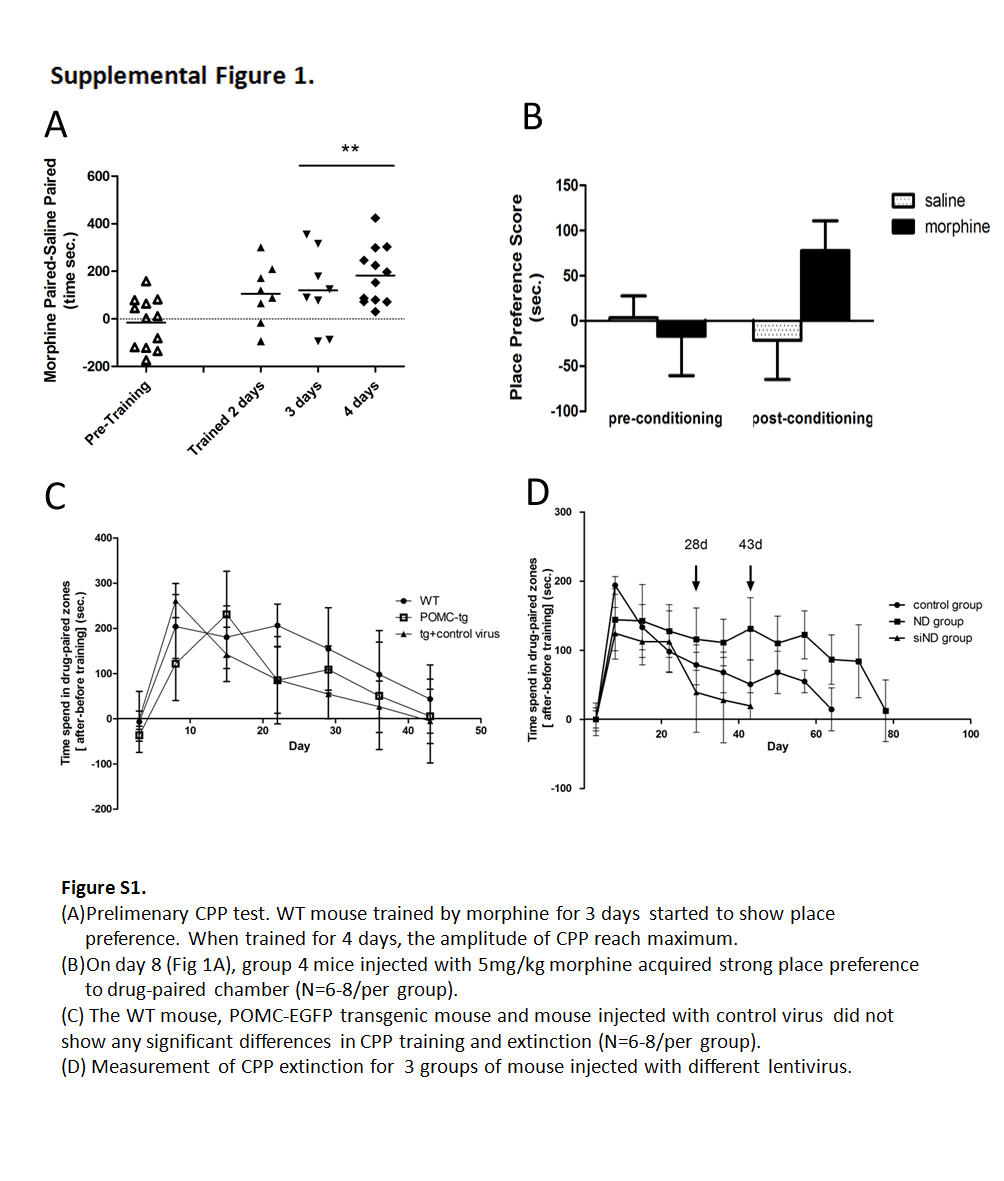

Supplement: S1 Fig — (A) Prelimenary CPP test. WT mouse trained by morphine for 3 days started to show place preference. When trained for 4 days, the amplitude of CPP reach maximum. (B) On day 8 (Fig 1A), group 4 mice injected with 5mg/kg morphine acquired strong place preference to drug-paired chamber (N = 6-8/per group). (C) The WT mouse, POMC-EGFP transgenic mouse and mouse injected with control virus did not show any significant differences in CPP training and extinction (N = 6-8/per group). (D) Measurement of CPP extinction for 3 groups of mouse injected with different lentivirus. (TIF) [file pone.0153628.s001.tif]

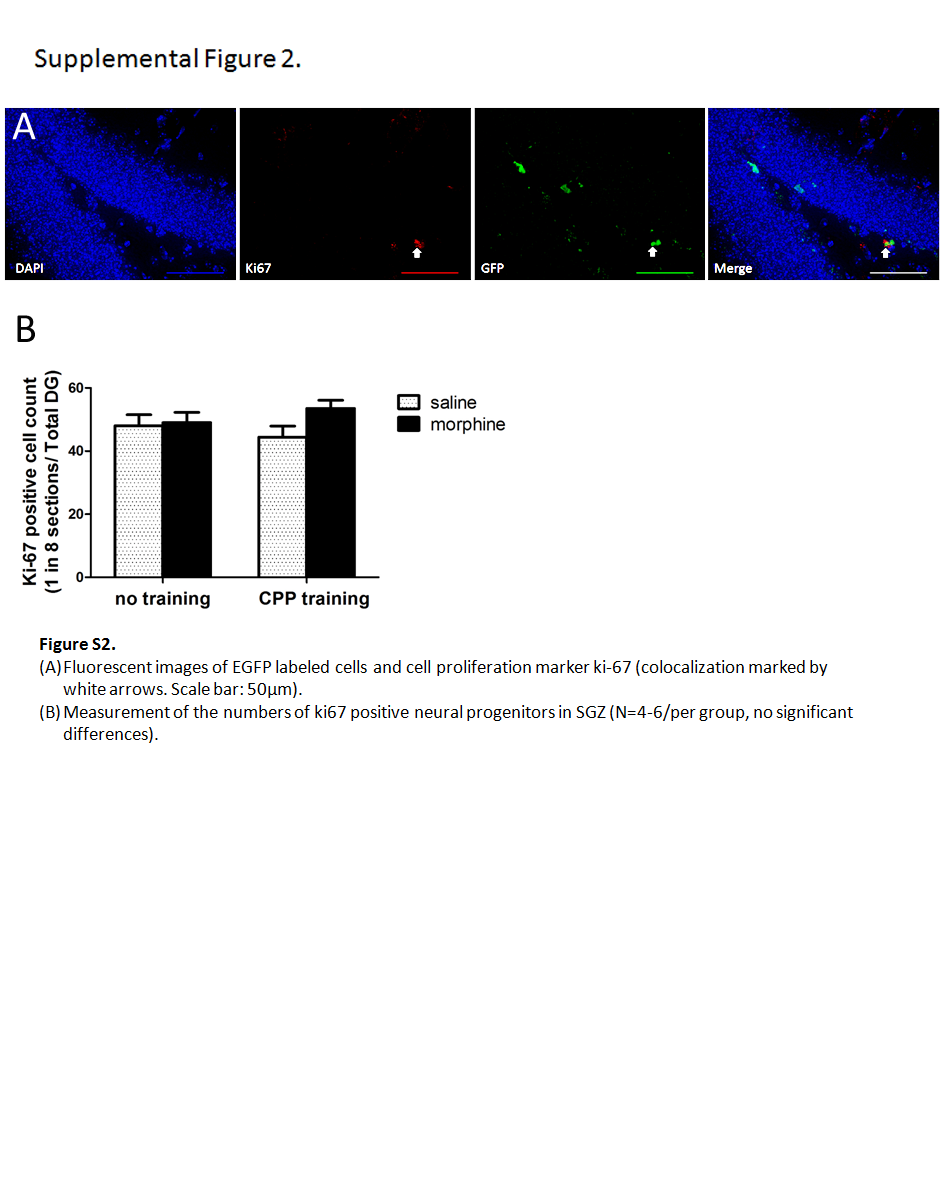

Supplement: S2 Fig — (A) Fluorescent images of EGFP labeled cells and cell proliferation marker ki-67 (colocalization marked by white arrows. Scale bar: 50μm). (B) Measurement of the numbers of ki67 positive neural progenitors in SGZ (N = 4-6/per group, no significant differences). (TIF) [file pone.0153628.s002.tif]

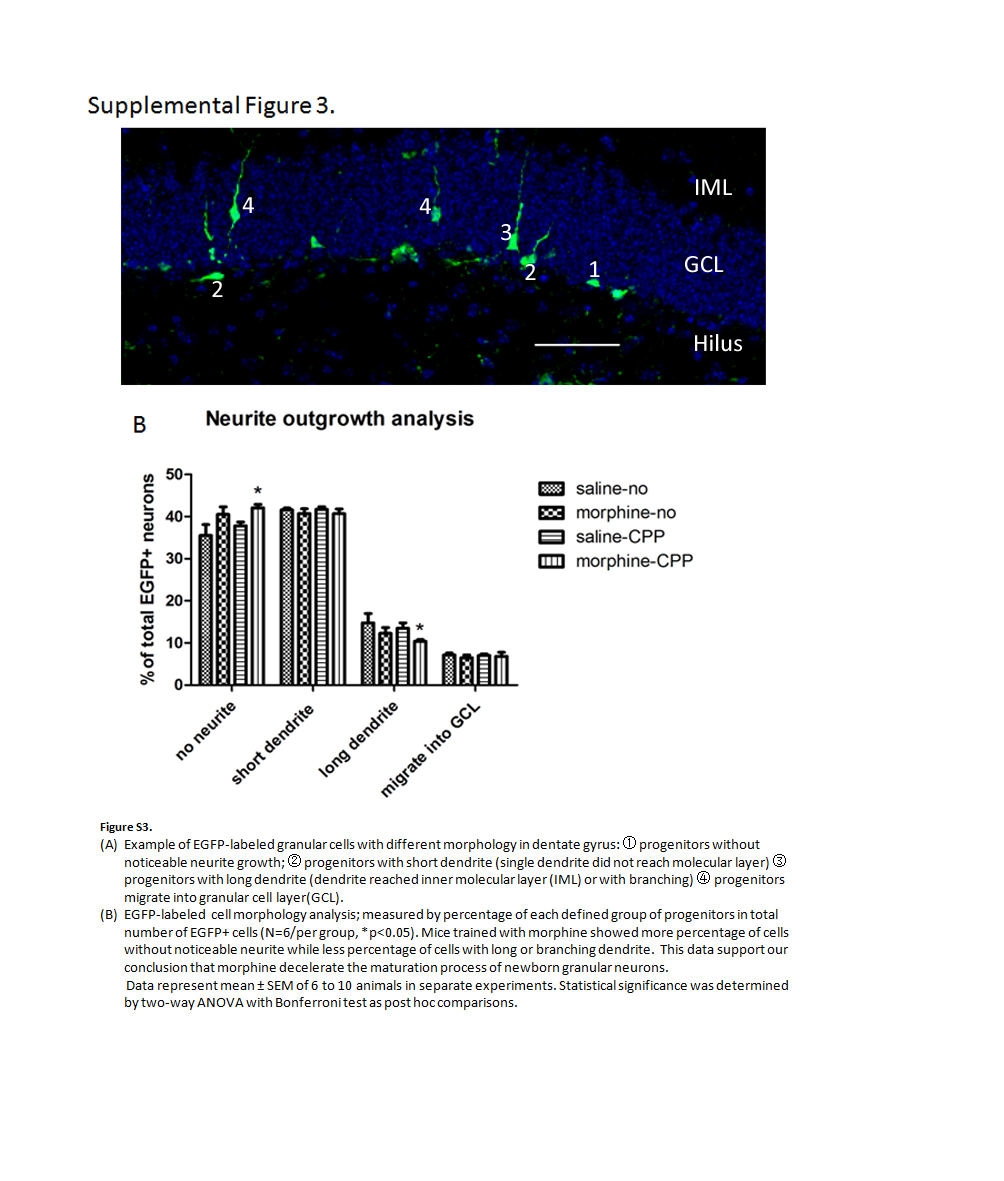

Supplement: S3 Fig — (A) Example of EGFP-labeled granular cells with different morphology in dentate gyrus: ① progenitors without noticeable neurite growth; ② progenitors with short dendrite (single dendrite did not reach molecular layer) ③ progenitors with long dendrite (dendrite reached inner molecular layer (IML) or with branching) ④ progenitors migrate into granular cell layer (GCL). (B) EGFP-labeled cell morphology analysis; measured by percentage of each defined group of progenitors in total number of EGFP+ cells (N = 6/per group, *p<0.05). Mice trained with morphine showed more percentage of cells without noticeable neurite while less percentage of cells with long or branching dendrite. This data support our conclusion that morphine decelerate the maturation process of newborn granular neurons. Data represent mean ± SEM of 6 to 10 animals in separate experiments. Statistical significance was determined by two-way ANOVA with Bonferroni test as post hoc comparisons. (TIF) [file pone.0153628.s003.tif]

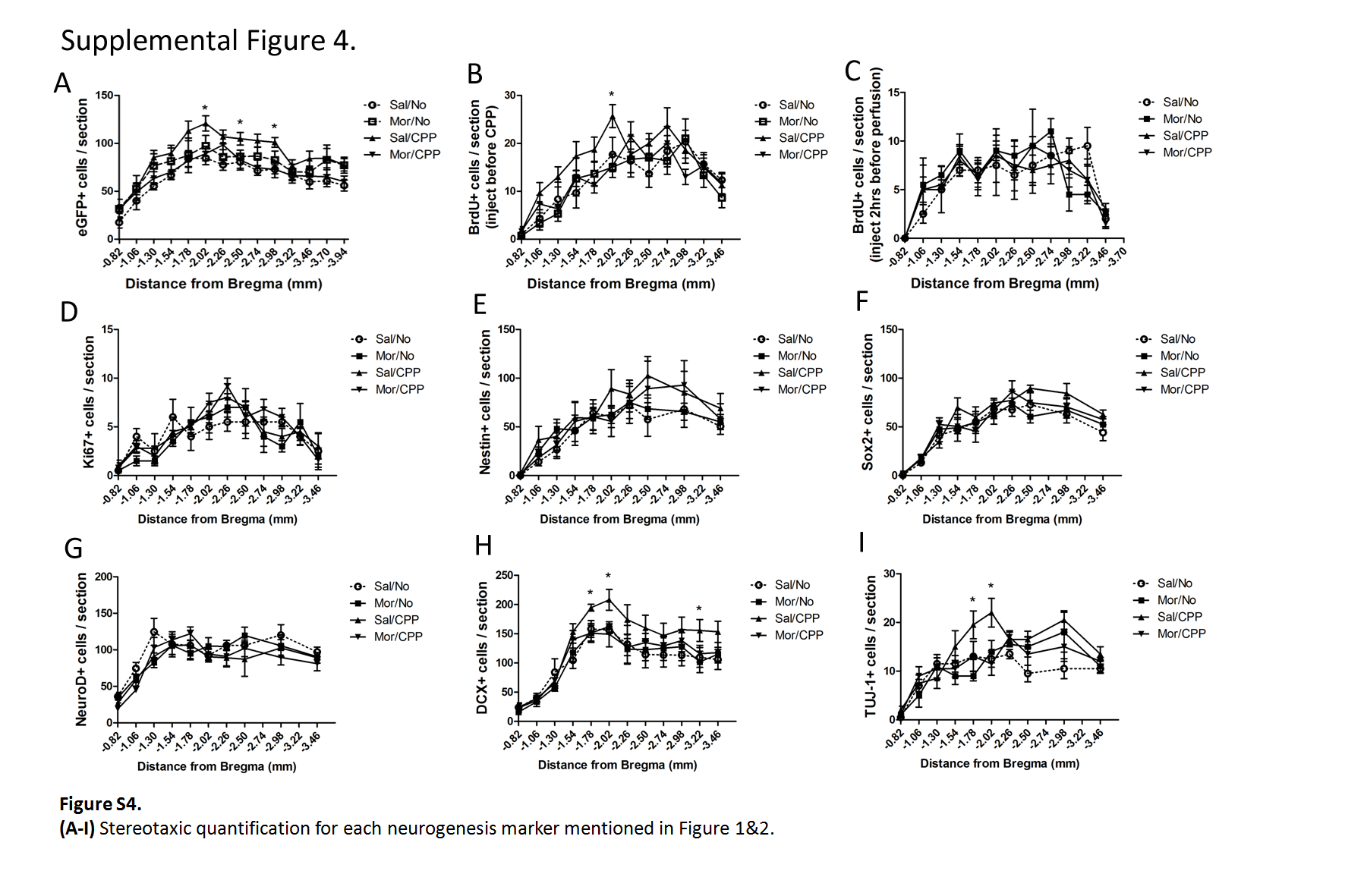

Supplement: S4 Fig — (A-I) Stereotaxic quantification for each neurogenesis marker mentioned in Figs 1 and 2. (TIF) [file pone.0153628.s004.tif]
